# Present Day Faith in Sputum Examination in Its Relation to Pulmonary Tuberculosis

**Published:** 1914-02

**Authors:** A. B. Elkin

**Affiliations:** Atlanta, Ga., Lecturer on Physical Diagnosis, Atlanta Medical College


### PRESENT DAY FAITH IN SPUTUM EXAMINATION IN ITS RELATION TO PULMONARY TUBERCULOSIS.

By A. B. Elkin, M. D., Atlanta, Ga.
Lecturer on Physical Diagnosis, Atlanta Medical College.

The examination of the sputum from a patient suspected of having Pulmonary Tuberculosis reveals the presence or absence of the tubercle bacilli in that specimen—nothing more.

The faith many have in this analysis when confronted with the problem of making a diagnosis, puts it on a plane undeserved. Several negative reports, particularly if the patient be robust and well-nourished in appearance, are more than apt to supplant the diagnostic value of physical signs if we are not well guarded against the mental effect of such reports.

Persistent search for the bacilli in all stages of the disease leads one to doubt its value save for confirmation, effect on the patient and a desire on the part of the examiner to make as complete an examination as possible.

The title of this paper may be criticised because of our constant demand for laboratory work, together with the fact that a positive report makes the diagnosis clear. Its purpose, however, is to attack the "negative reports" regardless of the condition of the patient or stage of the disease.

Unlike the abdomen, the chest seldom sounds the cry of distress unless there are present well defined physical signs, and we are positive these signs can persist without the confirmation of a microscope.

Considering the pathology of well advanced cases of tuberculosis one would naturally expect to find the bacilli in great numbers in a specimen coughed up, but this does not hold good in all cases, even where we are sure from the physical signs that the patient is well advanced. The following case history is of interest supporting this contention.

A white woman, aged forty-five, was admitted to the Waverly Hill Sanatorium, Louisville, Ky., with a diagnosis of "far advanced" tuberculosis. All of the physical signs of this stage were present, including a large cavity at the right apex. She had moist mucous rales extending posteriorly on both sides to the seventh dorsal spinae, and coughed almost constantly.

The characteristic symptoms, such as temperature, night sweats and marked amaciation were present. A competent pathologist made repeated examinations and was never able to demonstrate the germ, except in one instance, and then there were but very few discovered.

On the other hand, I am aware that the very early cases, showing very few of the physical signs sometimes show great numbers of the bacilli in their sputum. Such is the inconsistency of medicine. I am also aware that the majority of advanced cases will show the bacilli in their sputum, but I am satisfied that too much confidence is placed in this analysis by some who are "on the fence" in their diagnosis. In fact, how often is it that the final analysis comes with the sputum examination? How often, when the diagnosis of tuberculosis is not desirable that the sputum examination must shoulder the burden, and then with one or two examinations? How often is it that physical signs are detected, and a negative report of the sputum changes the complexion of things?

It is a conceded fact that in the vast majority of cases, physical signs, regardless of the degree, persisting for any length of time and unaccounted for, mean tuberculosis. It is the opinion of many that every person thirty years of age has, at one time or another, had an active infection. Why not then give the patient the benefit of the doubt and treat these cases as if they were positively tubercular.

The diagnosis of incipient tuberculosis is not always an easy matter. The slightly prolonged expiration or the presence of but few fine rales, are not always easily detected, but when we do detect them, why should we then put the burden of proof on the mircroscope, when probably the patient is not even coughing.

Tuberculosis is becoming more and more a sociological problem. The activity shown by the state and municipal organizations are educating the people to where they now welcome fresh air rather than shut it out, so that treating the disease is not the proposition it was formerly.

Apparently cured or arrested cases have so advertised the possibilities of present day methods to the public, that the people now realize that a diagnosis of tuberculosis does not sign a death warrant. Considering these facts, and with statistics before us, showing that cases actually die without ever showing the germ in their sputum, I believe it is a mistake to depend at all on the examination of sputum so long as the reports are negative, and physical signs are present.

